# Enhancing banknote authentication by guiding attention to security features and manipulating prevalence expectancy

**DOI:** 10.1186/s41235-021-00341-x

**Published:** 2021-11-13

**Authors:** Frank van der Horst, Joshua Snell, Jan Theeuwes

**Affiliations:** 1 De Nederlandsche Bank (DNB), Amsterdam, The Netherlands; 2 Department of Experimental and Applied Psychology Vrije Universiteit, Amsterdam, The Netherlands; 3 Institute of Brain and Behavior Amsterdam (iBBA), Amsterdam, The Netherlands

**Keywords:** Attention, Decision-making, Gist, Vision, Cueing, Authentication, Banknotes, Counterfeits

## Abstract

All banknotes have security features which are intended to help determine whether they are false or genuine. Typically, however, the general public has limited knowledge of where on a banknote these security features can be found. Here, we tested whether counterfeit detection can be improved with the help of salient elements, designed to guide bottom-up visuospatial attention. We also tested the influence of the participant’s a priori level of trust in the authenticity of the banknote. In an online study (*N* = 422), a demographically diverse panel of Dutch participants distinguished genuine banknotes from banknotes with one (left- or right-sided) counterfeited security feature. Either normal banknotes (without novel design elements) or banknotes that contained a salient element (a pink rectangular frame) were presented for 1 s. To manipulate the participant’s level of trust, trials were administered in three blocks, whereby at the start of each block, participants were instructed that either one third, one half, or two thirds of the upcoming banknotes were counterfeit (though the true ratio was always 1:1). We hypothesized (i) that in the presence of a salient element, counterfeits would be better detected when the location of the salient element aligned with the location of the counterfeited security feature—i.e. that it would act as an attentional cue; and (ii) that this effect would be stronger with lower trust. Our hypotheses were partly confirmed: counterfeit detection improved with ‘valid cues’ and decreasing trust, but the level of trust did not modulate the cueing effect. As the overall detection performance was rather poor, we replicated the study with a sample of university students (*N* = 66), this time presenting stimuli until response. While indeed observing better overall performance, all other patterns were replicated. Our results provide evidence that attention can be guided to enhance banknote authentication.

## Introduction

Typically, people accept banknotes as change from another person or at a point-of-sale without consciously verifying authenticity (Klöne et al., 2019). Reasons for not checking authenticity are that counterfeit rates are extremely low, and that people trust the retailer (van der Horst et al., 2017a). Indeed, authentication may take place in a limited number of cases; for example, when the cash handler has encountered counterfeit banknotes before, or when the paper of a particular note feels somewhat unusual. Also, when one does not trust a particular transaction (e.g. an online purchase involving cash) one may check the authenticity of the banknote. A more practical constraint is that the general public has little knowledge of how to authenticate banknotes. On average, a person can mention two security features, but does not know what these features look like exactly, and where on a banknote these features may be found (van der Horst et al., 2017a). For instance, 69% of the general public knows that a euro banknote contains a watermark, but only 6% knows what image the watermark depicts (De Nederlandsche Bank, 2021a, 2021b). The next most known security feature is the hologram foil, mentioned by 39% of the public. The emerald number can be recalled only by 2% of participants.

As a consequence, a good deal of counterfeited banknotes goes undetected. To illustrate, van der Horst et al. (2017b) reported that around one in every five counterfeits is missed, in spite of the fact that participants were actively authenticating and were granted all the time they needed for this authentication task. It would not seem unreasonable to assume that the proportion of undetected counterfeits must be decidedly higher in everyday life, where cash handlers are not explicitly instructed to authenticate.

**Fig. 1:**
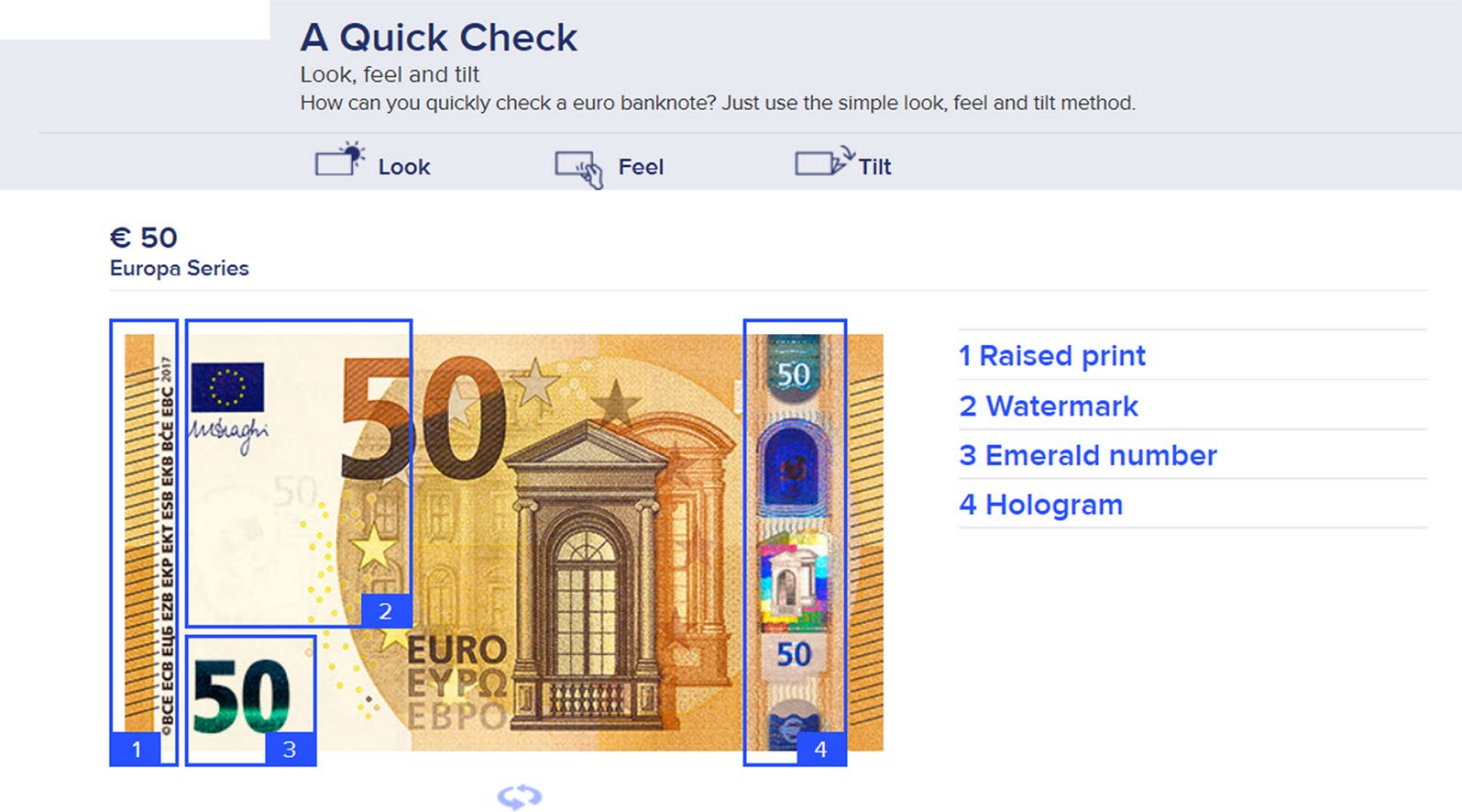
Instructional image on how to check the most prominent security features of a EUR 50 banknote quickly. Source: DNB website (www.dnb.nl/echtofvals)

Yearly, the Eurosystem removes around 560 thousand counterfeits from circulation (out of a total of 24 billion banknotes; ECB annual report 2019). For an overview of the most prominent public security features, as indicated by the Nederlandsche Bank (DNB), see Fig. 1.

Yet another reason for not checking the authenticity of a banknote may be that the authentication process itself would constitute a socially awkward or uncomfortable situation—all the more fuelled by the fact that aforementioned lack of knowledge would likely make the authentication process a long one. If cash handlers were able to authenticate banknotes more quickly and covertly, it may well be that fewer counterfeits would go unnoticed. Additionally, if banknotes were authenticated more easily, perpetrators may be less inclined to use counterfeit banknotes in the first place.

In short, members of the public are rarely inclined to check a banknote for its authenticity, but when they do, they lack the capability to do it properly. Here we investigated whether counterfeit detection can be improved with the addition of novel, salient visual elements, designed to guide visuospatial attention to critical locations. Additionally, we assessed the impact of one’s a priori trust on attentional orienting.

Our hypotheses were guided by two distinct fields of study. The attention literature led us to reason that a counterfeited security feature should be detected more readily when attention is directed to the security feature’s location. One way to ensure that attention is directed to a critical location is to introduce a visually salient element near the location of the security feature such that attention is captured towards the critical location in a bottom-up way (e.g. Theeuwes, 2010; Wolfe et al., 2003). The hypothesized beneficial effect on counterfeit detection performance of having a salient element near a security feature, would be analogous to an attentional cueing effect (Posner, 1980). With respect to one’s a priori of trust, we reasoned that lower levels of trust would increase overall performance (due to increased effort). We were largely agnostic with respect to interactions between trust and cue validity. On the one hand, one might argue that increased effort (induced by low trust) would cause stronger attentional orienting and consequently stronger capture by salient design elements. On the other hand, an increased contribution of top-down attention might reduce the strength of bottom-up attentional capture. Let us now turn to these attentional dynamics.

### Attentional processes in counterfeit detection

Cash transactions at a point-of-sale are generally performed quickly and automatically (van der Horst & Matthijsen, 2013). People do not give themselves time, or might feel embarrassed when scrutinizing the banknote (De Heij, 2017).

To authenticate a banknote properly, a good strategy is to direct attention to the security features. Attentional orienting can proceed in a bottom-up and top-down manner. Bottom-up attention is usually deployed reflexively due to the characteristics of the scene and stimulus saliency (e.g. Theeuwes et al., 2003), although the capture of attention can be prevented via an inhibitory mechanism that suppresses the salient stimulus (Luck et al., 2021). Top-down attention, which is thought to underly that inhibition, is usually deployed voluntarily in line with one’s tasks and goals (Egeth & Yantis, 1997). However, top-down authentication of banknotes is likely hampered by the handler’s aforementioned lack of knowledge.

It would therefore be ideal if security features were to capture attention in a rapid bottom-up manner (e.g. Theeuwes, 2019). It is worth noting that there has recently been a marked rise of simplified counterfeits without (mimicked) security features (Deutsche Bundesbank, 7–8-2020), suggesting that if attention were directed immediately and briefly to the relevant location on a banknote this could improve counterfeit detection. This underlines the importance of guiding banknote users’ attention to security features.

It may come as no surprise that saliency is a well-known concept among developers of banknote security features. For instance, nano-optic display technology features deliver a sense of movement, 3D depth, and multiple colours. According to manufacturers these technologies enable a wide array of custom design options to both capture and hold the user’s attention as they inspect and authenticate a banknote (16-11-2020, https://www.nanosecurity.ca/banknote-security/). However, to date there is no scientific dissemination about the effectiveness of security feature saliency. Furthermore, one must take into account the possibility that with increased saliency of one security feature, attention may increasingly be directed away from other security features. One challenge is thus to achieve optimally balanced saliency across features—a challenge enlarged by the fact that features differ from each other in terms of shape and size.

A potential solution—and the focus of this study—is to display a *single* type of salient element near each security feature. As such, the security features themselves can stay as they are, while the novel salient design element may become an established marker for areas worthy of inspection.

Although there is a lot of research suggesting that attention can be guided with the help of salient visual elements (e.g. Theeuwes, 2010), we must nonetheless be aware of one potential constraint. It is known that the most salient elements in a display typically receive attention first—irrespective of whether they are relevant or irrelevant (Wang & Theeuwes, 2020). Hence, if the salient element is at the same location as the security feature—as in the case of, say, a pink frame around the banknote’s emerald number—attention would be at the right location; but would it predominantly be directed to the pink frame, or to the emerald number itself? In the former scenario, the salient element would be helpful in roughly guiding attention (e.g. attention would be oriented to the right quadrant of the banknote), whilst interfering at a more detailed level (e.g. attention would be focused on the pink frame rather than on what is in the frame).

We chose the colour pink (desaturated red) for the frame, because of its saliency. In an experiment conducted by Drelie Gelasca et al. (2005) participants had to rank 12 colours in terms of saliency. The colours that had much more hits were red, yellow, green and pink. Those of lower saliency seemed to be light blue, maroon, violet and dark green. Also, in a colour experiment in which two groups searched for desaturated targets among saturated and white distractors, the conclusion was that the pink and peach targets have an advantage over the green, blue, and purple targets concerning reaction times (Kuzmova et al., 2008).

### The impact of trust

As noted earlier, we expect that persons who have high trust in the authenticity of banknotes, for example because they assume that the counterfeit rate is low, perform worse than persons who expect a higher counterfeit rate. This hypothesis is based on the ‘prevalence-effect’. Observers tend to miss a disproportionate number of targets when these targets are rare (Wolfe & Van Wert, 2010). In everyday life, the prevalence of counterfeits is very low. The general public mentions this as an important reason for not authenticating (Klöne et al., 2019).

Lau and Huang (2010) found that the prevalence effect depends on past experience, not on future prospects. In their study, participants were told either that targets would be frequent (50%) or rare (10%), and both these instruction types were provided in settings where the true prevalence was either 50% or 10%; (hence, prevalence and *the expectancy thereof* were orthogonally manipulated). As it turned out, the error rate depended not on the instructions given but on the true target prevalence of the blocks. However, it might have been the case that participants simply did not believe the instructions (i.e. that expectancy was not successfully manipulated).

In fact, other research suggests that both target repetition and target expectancy play a role in the prevalence effect (Godwin et al., 2016). In the study of Godwin et al., one group of participants searched for low and high-prevalence targets of one particular colour throughout the experiment, while another group searched for one target colour on high-prevalence slides and a different target on low-prevalence slides. As such participants received differential levels of target repetition across the lower and higher-prevalence targets. An effect of prevalence emerged in both groups, although it was weaker in the single colour condition than it was in the alternating-colour condition, suggesting that both target repetition and target expectancy play a role in the prevalence effect.

Previous studies have shown that prevalence expectancy can simply be influenced by task instructions. For example, in their investigation of lesion detection on chest radiographs, Nocum et al. (2013) found that expectations of a higher abnormality-prevalence rate, as induced by instructions, impacted doctors’ perceptual sensitivity and visual search patterns, even though observers received the same stimulus material.

In the current study, we manipulated the expectancy of prevalence, which was assumed to affect top-down attention, and manipulated the presence or absence of a salient element around security features, which was assumed to affect bottom-up attention. The manipulation of expectancy is particularly important as it is one of the underlying factors of the trust one has in the payment system. The rationale is that people who have low trust in the authenticity of banknotes expect that the counterfeit rate is relatively high are more likely to invest more effort in authentication and thereby, to enhance authentication (van der Horst, et al., 2020a).

### The present study

To summarize the above, typically the general public does not authenticate banknotes because they trust the banknote to be genuine and because they have insufficient explicit knowledge about which locations on the banknote inform its authenticity. Therefore, in this study, we examined whether salient elements around security features may help the public in authenticating a banknote at a quick glance. It is important to determine whether authenticating can be done rapidly because cash transactions typically occur within a very brief time frame (van der Horst et al., 2020b). We hypothesized that displaying a pink frame around a counterfeited security feature would lead to better counterfeit detection. This manipulation is to some extent analogous with the classic Posner exogenous cueing paradigm (Posner, 1980), in which targets are typically detected faster and more accurately when a cue is valid than when it is invalid.

Importantly, we did not instruct our participants on the existence and location of security features, as the general public is not trained either. Below it will be seen that overall detection scores were indeed not very high. However, our focus is not the performance per se, but the difference between having a salient element near to versus away from, the counterfeited feature, thought to operate as a valid versus invalid attentional cue, respectively. By directing the participants attention to a counterfeited feature, we expect to improve their ability to categorize the banknote as counterfeit.

## Method

### Participants

In order to have a representative sample of the general public in the Netherlands, we made use of the LISS panel (longitudinal Internet Studies for the Social Sciences) run by CentERdata at Tilburg University. This panel is representative of the general population in the Netherlands and comprises around 5000 households in the Netherlands. We aimed for a net sample of 400 participants, but in total 451 participants participated in the experiment. The panellists were 16 years and older. They received a small monetary compensation (EUR 7.50, real money) for their expenses (internet use and time).

### Design

The experiment followed a 3 × 3 × 4 within-subjects design, with the following factors: *Cue* (left, right, none); *Trust* (high, mid, and low, corresponding to low, mid, high counterfeit expectancy); *Authenticity* (counterfeit element left, counterfeit element right, genuine, genuine); genuine is mentioned twice to have the same number of genuine versus counterfeit trials.

### Stimuli

The test set consisted of images of genuine euro banknotes that were taken out of circulation and visually altered (counterfeit) versions of the same banknotes. We created counterfeits by replacing a single genuine security feature by a cut-out of a counterfeited security feature. There were two types of counterfeited security features: the hologram (silvery stripe) that is positioned at the right side of the banknote and the emerald number that is positioned at the left side of the banknote, corresponding to the counterfeit element right and left conditions. The cut-outs were obtained from counterfeits taken out of circulation by De Nederlandsche Bank. We used cut-outs of simple ink-jet counterfeits instead of the ones printed with offset techniques, as these are the most prevalent. According to DNB’s national counterfeit analysis centre, the counterfeited elements in our test set were of average mimicking quality, which means that a counterfeited element can be noticed visually by the average person when attention is directed to it.

Additionally, for all banknote stimuli we created versions with a salient pink rectangle framing either the left or right-sided security feature. Because the hypothesized effects of having a salient element near to or away from a counterfeited feature are interpreted as attentional cueing effects, versions of counterfeited notes with salient element at the same versus different location as the counterfeited feature represent the Valid Cue and Invalid Cue conditions, respectively. We chose the colour pink because it is rated as a particularly salient colour (e.g. Drelie Gelasca et al., 2005; Kuzmova et al., 2008).

**Fig. 2:**
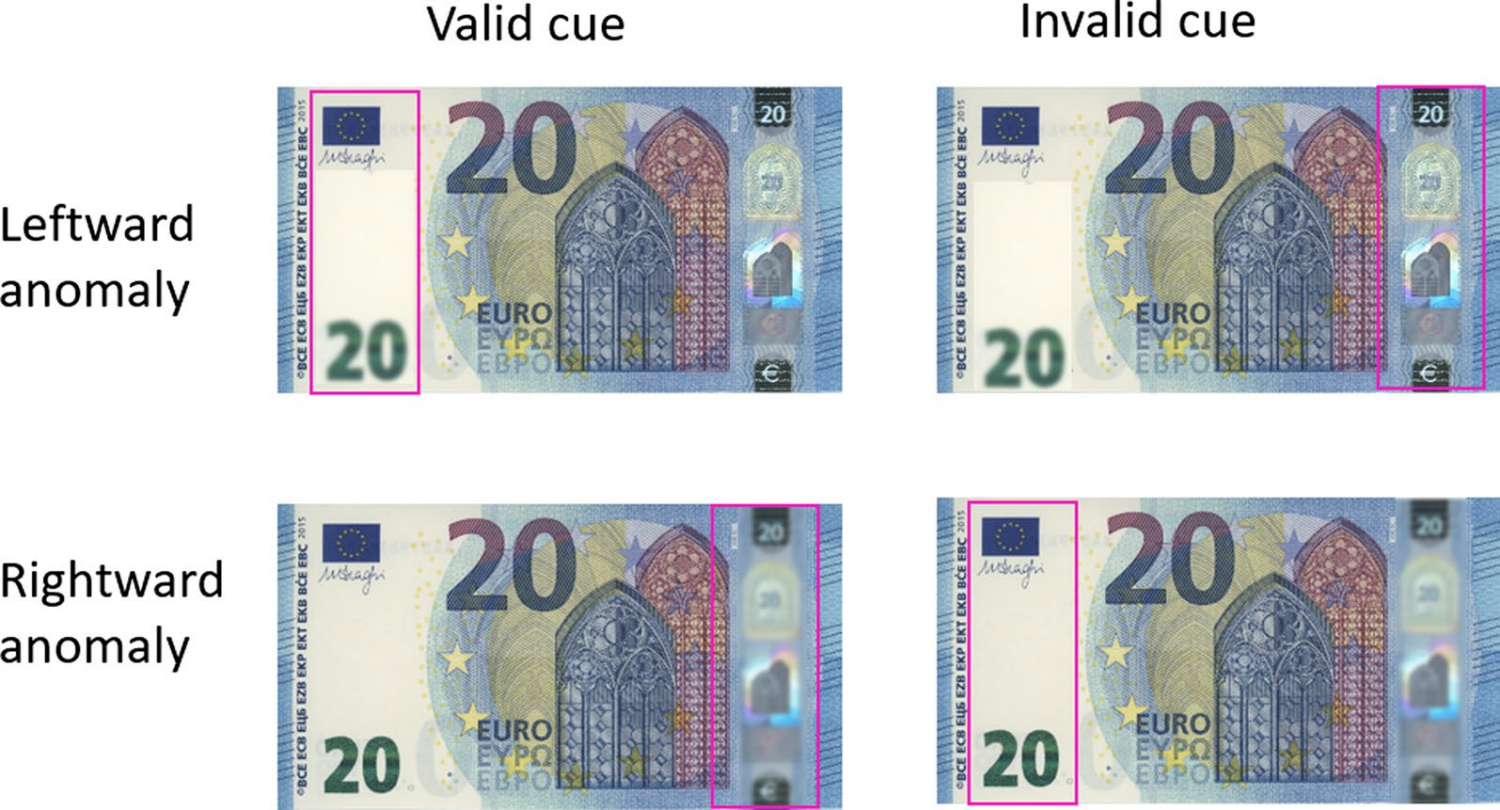
Examples of manipulated banknotes that are part of the test set. The banknotes on top contain a counterfeited emerald number: top-left with a pink cue around the counterfeited emerald number; top-right with the pink cue around a genuine hologram. At the bottom, banknotes with a counterfeited hologram: left-bottom a pink cue around the counterfeited hologram; right-bottom with a cue around a genuine emerald number. The two banknotes on the left are validly cued (the cue is located near the feature that is counterfeited). The two banknotes on the right are invalidly cued: the cue is near a genuine feature, while the counterfeited feature is at the other side

We used both EUR 20 and EUR 50 banknotes (denomination not being considered an experimental factor). The complete stimulus set consisted of 24 images, i.e. 2 Authenticity (genuine/counterfeit) × 3 Cue (left/right/no cue) × 2 Security feature (hologram/emerald number) × 2 Denomination (EUR 20/50). Denominations EUR 20 and 50 were used because these are by far the most used *and* counterfeited ones (press release DNB, 22 January 2021). The denominations EUR 20 and EUR 50 were manipulated according to the same method described above. Figure 2 shows examples of manipulated banknotes.

Clearly, the proportion of genuine and counterfeit banknotes in the test set (1:1) is quite different from the probability of encountering a counterfeit in real life, which is roughly 0.003% (ECB, 2019). In addition, one’s perceived likelihood that one will receive a counterfeit does not directly reflect real-world prevalence either. Instead, we would argue that counterfeit expectancy is a function of immediate context, and that the subjective biases that stem from this context are much more variable than real-world counterfeit prevalence. It is these variations in subjective prevalence expectancy that are studied here.

### Procedure

Participants were invited to perform the test online on their own computers. For this reason, there was little control over the degrees of visual angle of our stimuli.

**Fig. 3:**
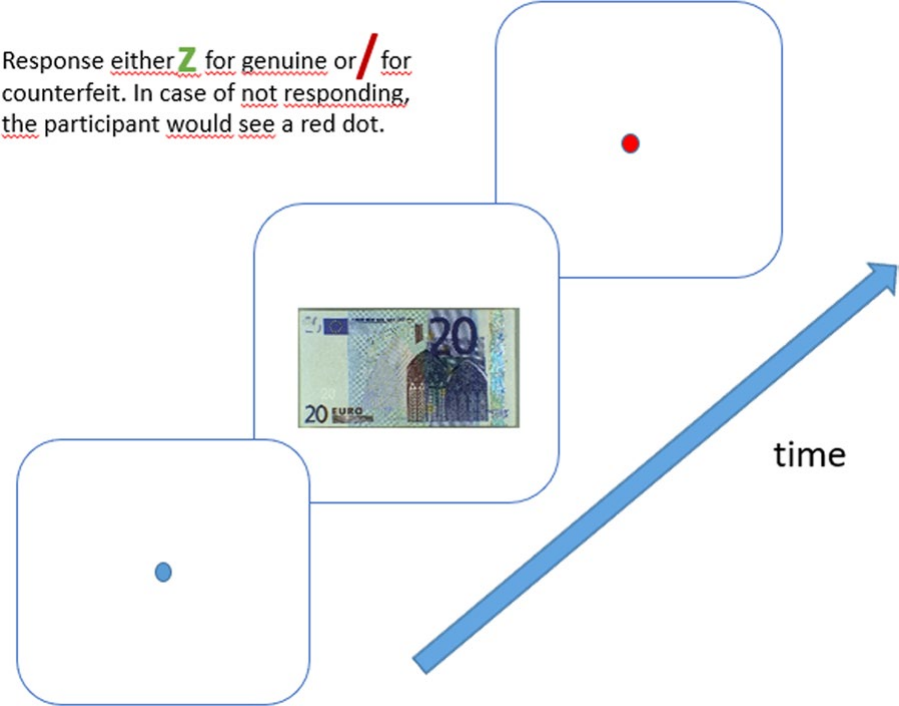
Example of a trial. Each trial started with a fixation dot in the centre, for 500 ms, followed by a banknote (either EUR 20 or EUR 50, either genuine or counterfeit, either with a cue or not). The display duration was 1000 ms. The information regarding the ratio of counterfeits was varied between blocks. If participants failed to press a key within 4,000 ms from stimulus onset, the trial was logged as a time-out

In the instructions participants were told that DNB wanted to test some design elements and that therefore a pink rectangle could be seen on the majority of banknotes. However, according to the instructions these new design elements would have no relation to whether the note was genuine or not. Next, participants were informed that banknotes would be presented for one second. They were instructed to authenticate the banknotes by typing a ‘z’ for genuine and ‘/’ for counterfeit after the banknote was presented. They were instructed to respond as accurately as possible. They had a maximum of 4000 ms to respond (after which the response would be considered an ‘error’). Banknotes were presented centrally, albeit with minor jitter (ranging up to 40 pixels) in the banknote’s *x* and *y* coordinates, so as to prevent participants from developing oculomotor strategies. An overview of the trial procedure is shown in Fig. 3. To get acquainted with the procedure, participants performed 12 practice trials that were not included in the data analyses.

The participants’ trust in banknote genuineness was manipulated between blocks. All 24 images were presented three times, in three blocks (presented in random order for each participant). Every time before the start of a block, participants were informed on the expected ratio between genuine and counterfeits for the upcoming block: (i) two out of three, (ii) even, and (iii) one out of three. In reality, the genuine vs. counterfeit ratio was always 1:1.

At the end of the experiment, participants received feedback regarding their performance: a percentage correct was provided for all three blocks. Participants were invited to fill in a short survey for demographics, colour blindness and cash experience in working life (for the purpose of post hoc analyses). The experiment took approximately 10 min.

## Results

All trials with a time-out were removed. In case this resulted in removing more than a third of a participant’s trials, the data of this participant were removed altogether, as this indicates that the participant was not able to perform the task properly. In total, 29 participants were removed, constituting 9.1% of the data. The results of the remaining 422 participants were used.

To reiterate, the experiment included the following factors: Cue Validity (*valid* vs. *invalid cues*) and Trust (*low, mid-* and *high* levels of trust). These variables allowed us to rely, in part, on measures derived from Signal Detection Theory (SDT). The ability to discriminate genuine banknotes from manipulated banknotes is called sensitivity (d’), which can be estimated by deducting the z-transformed probability of false alarms (i.e. incorrectly classifying a genuine banknote as being counterfeit) from the z-transformed probability of hits. A d’ score of 0 corresponds to a complete inability to distinguish genuine banknotes from counterfeits. According to Raymond (2017), a d’ of 1.25 represents decent sensitivity in banknote authentication. The maximum d’ score that can be obtained in this study is 3.92.

Importantly, while d’ can be calculated when inspecting main effects of Trust (i.e. irrespective of cueing condition), this is not the case when inspecting main effects of Cue Validity (i.e. irrespective of level of trust). This is because the cue valid and invalid conditions solely contain counterfeit banknote trials (indeed, consider that there is no such thing as a validly cued genuine banknote), and therefore one cannot conjure a false alarm rate required for the calculation of d’. Hence, in all analyses that involved the Cue Validity factor, we simply relied on accuracy (the SDT-equivalent of which would be the hit rate, retrieved from counterfeit banknote trials). Our central analysis (reported in "Central analyses" section) was thus a 2 × 3 repeated measures analysis of variance (ANOVA) with Cue Validity and Trust as factors, and accuracy as dependent variable.

We nonetheless also analysed Trust in isolation ("Verifying the manipulation of trust" section), as we could retrieve not only d’, but also the response bias (i.e. the extent to which one response is more likely to be given than another), or β, when inspecting this variable separately. The β measure, calculated by dividing the z-transformed probability of hits by the z-transformed probability of false alarms, provides an important verification of the effectiveness of our Trust manipulation. That is, if participants took the block instructions to heart, we expected them to have marked a larger portion of genuine banknotes as counterfeit upon being warned for a high counterfeit prevalence (although actual prevalence did not vary across conditions). At the same time, we may expect them to mark a low number of counterfeits as being genuine. Upon being warned for a low counterfeit prevalence, we would expect these patterns to be inversed. In short, if our Trust manipulation was indeed effective, we expect that β would be higher (i.e. more conservative) in the high-trust than in the low-trust condition.

### Verifying the manipulation of trust

Repeated measures ANOVAs were used to analyse main effects of Trust on d’ and β. Overall, sensitivity did not increase linearly with a decrease in Trust (*F*(2,421) = 2.131, *p* = 0.119). We did, on the other hand, observe a numerical effect of Trust on β that approached significance: (*F*(2,421) = 2.437, *p* = 0.088), with a more conservative response strategy in the high-trust than in the low-trust condition: i.e. lower levels of trust aided counterfeit detection, but, at the same time, caused a higher proportion of false alarms. From these results we conclude that the way in which we manipulated trust was effective.

### Central analyses

**Fig. 4:**
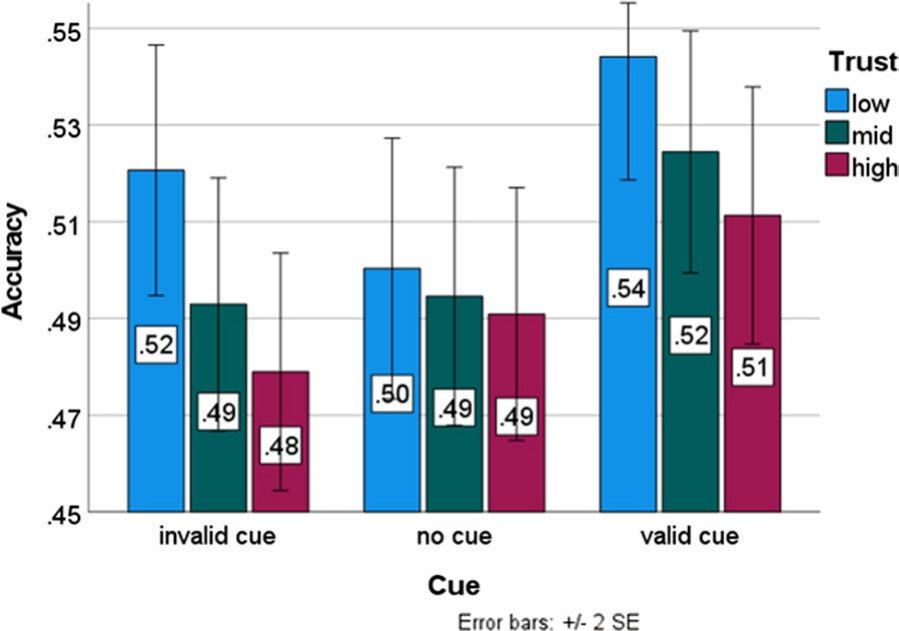
Average accuracy per level of trust (low, mid, high) and cueing condition. Both a low trust in the authenticity (i.e. a high expectancy on the number of counterfeits) and valid cueing led to better performance. Error bars depict 95% confidence intervals

A repeated measures ANOVA was run with Cue Validity and Trust as factors and Accuracy as dependent variable. In line with our hypotheses, valid cues led to better accuracy than invalid cues: *F*(2,421) = 4.969, *p* = 0.007, *η*^2^*p* = 0.012. Again, we also observed a main effect of Trust (*F*(2,421) = 3.916, *p* = 0.020, *η*^2^*p* = 0.01), with better counterfeit detection at lower levels of trust; (however, given the absence of effects in d’ and the reversed effect for genuine banknotes, as reported in "Verifying the manipulation of trust" section, it can be argued that this particular effect reflects a shift in β, rather than a change in overall performance). Trust did not modulate the effect of Cue Validity: *F*(4,421) = 0.621, *p* = 0.648. Figure 4 shows the average scores for the nine conditions.

Evidently, overall authentication performance was quite poor in this population sample. In order to determine whether the task was too difficult, we calculated the average sensitivity scores in the no-cue condition, since this condition provides a baseline (without novel design elements) and as such can be compared to the study of van der Horst et al. (2020a, 2020b). We observed a sensitivity of *d*’ = 0.386, which is indeed decidedly lower than the sensitivity *d*’ = 1.05 observed in the study of van der Horst et al. (2020a, 2020b). Although overall sensitivity was quite low, it was significantly above chance-level (*t*(421) = 11.274, *p* ≤ 0.001). It is also worth noting that the low sensitivity was unlikely to be driven by a lack of expertise: people who responded to have experience with cash in a professional setting did not perform differently from the others (*t*(420) = 1.269, *p* = 0.205).

We reckon that recognizing a single fake element in an image of a banknote that is exposed for only one second might be difficult for non-trained members of the general public. Crucial in this regard is the fact that the salient design element, when acting as a valid cue, significantly improved performance.

We wanted to examine if the observed effect of cueing would also hold if the task was less difficult. For this reason, we decided to run the same experiment with a group of 66 psychology students and this time presenting the images of the banknotes until response. The results of this replication experiment are presented in the Appendix. Importantly, while the overall performance in this population sample was indeed better, we replicated all effects of interest (the bias of participants increased with a lower trust in the authenticity of banknotes: *F*(2,66) = 3.639, *p* = 0.029). Just like the experiment with participants from the CenTErdata panel, we found main effects for accuracy per cueing validity (*F*(2,66) = 4.565, *p* = 0.012), *η*^2^*p* = 0.07 and trust (*F*(2,66) = 4.304, *p* = 0.015), *η*^2^*p* = 0.06 and no interaction between these factors: *F*(4,66) = 0.989, *p* = 0.414. In addition trust affected sensitivity scores adversely: *F*(2,66) = 4.103, *p* = 0.019.

## General discussion

The goal of this study was to investigate whether salient design elements, intended to direct attention to the location of security features, would aid banknote authentication accuracy. In our experiments, pink frames around a counterfeited security feature were expected to act as a cue, akin to attentional cues in classic tasks such as Posner’s cueing paradigm (1980). Similarly, a pink frame around a genuine security feature, when at the opposite side a counterfeited security feature was present, was expected to act as an invalid attentional cue. Participants were not instructed to react to these salient elements; they were only told that DNB wanted to test some new design elements. Across two experiments we confirmed our expectations. Banknotes with a salient element around the counterfeited feature location yielded better detection than banknotes with an ‘invalid cue’ (i.e. a salient element at a different location). These results provide a proof-of-concept that salient novel design elements can aid banknote authentication.

We also found that lower levels of trust aided counterfeit detection, but, at the same time, caused a higher proportion of false alarms ("Verifying the manipulation of trust" section). It is worth considering that although high counterfeit detection rates are undoubtedly beneficial, effectuating these by means of lowering trust would imply extensive examination processes (i.e. more false alarms) and likely less smooth functioning of the cash payments system. Central banks may want to consider this particular finding when they issue press releases informing the public about counterfeit prevalence. In relation to this, Lau and Huang (2010) have argued that instructions alone might not be very effective in reducing error rates in real-life low-prevalence contexts, such as airport baggage screening or counterfeit banknote detection. Instead these authors have argued for randomly distributing ‘pseudo-targets’. This would imply an artificial increase in prevalence, and the experience gained with such pseudo-targets would reduce the chance of missing actual targets. Applying this idea to the realm of banknote authentication, central banks might consider purposefully bringing counterfeits into circulation, which, upon being spotted and reported, would yield a reward. Naturally, discussions of the legal constraints surrounding such operationalizations of trust and prevalence are beyond the scope of this paper.

The average sensitivity or *d*’ in the no-cue (baseline) condition in the present experiment was 0.386. A d’ of 0 corresponds to a complete inability to distinguish genuine banknotes from manipulated banknotes; and, according to Raymond (2017), a *d*’ of 1.25 represents decent authentication sensitivity. Previous research (Van der Horst et al., 2020a, 2020b) showed a higher average sensitivity (*d*’ = 1.05) for the general public in a task similar to the present one (i.e. participants had to detect counterfeit banknotes that were presented for one second on a screen). There are however also important differences between the two experiments. Firstly, participants encountered novel design elements in the present study, which they ought to treat as being non-informative about the banknote’s authenticity. Secondly, in the present study counterfeit banknotes contained only one counterfeit element, the emerald number or the hologram. Lastly, the counterfeit quality may have differed between the studies. These factors possibly made the distinction between genuine and counterfeit banknotes smaller than in the study of van der Horst et al. (2020a, 2020b).

In our replication experiment with psychology students (*N* = 66) that saw the stimulus until response, overall performance was decidedly better (*d*’ = 1.73 in the baseline condition). The pattern of positive effects on counterfeit detection by validly cueing and low trust was also found in this replication experiment.

The present findings demonstrate a possible role for bottom-up saliency to aid banknote authentication.

One potential caveat, however, is that attending to one security feature (helped by a salient element) may come at the cost of not attending to another, equally important security feature. Further tests of our hypotheses may involve comparing the authentication of banknotes without pink frame, against banknotes with multiple pink frames (i.e. one around each security feature). If our claims hold, then the pink rectangles should facilitate quicker serial processing of all relevant locations on the banknote, and thus better performance as compared to banknotes without pink rectangles.

Lastly, while saliency should help in finding the security features, what to do next—i.e. how to use these security features for successful authentication—remains a challenge. Further research on making the security features more intuitive may thus be beneficial for counterfeit detection.

In conclusion, the present findings suggest that salient design elements may aid counterfeit detection. This cueing effect is also shown for perceptual sensitivity measures such as accuracy and d’ (Bashinski & Bacharach, 1980; Theeuwes & Van der Burg, 2007). Additionally, as low levels of trust positively impacted authentication, we posit that the general public would benefit from increased awareness about the existence of counterfeit banknotes.

## Figures and Tables

**Fig. 5:**
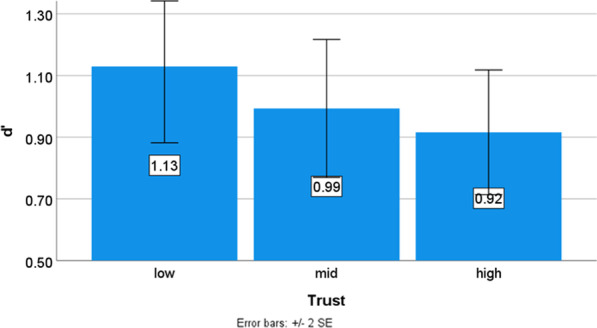
Average authentication sensitivity scores per condition of trust in the authenticity of a banknote (low, mid, high). Presentation time is until response. The sensitivity scores of participants significantly changed when the expectancy of the ratio of counterfeits was varied. Error bars depict 95% confidence intervals

**Fig. 6:**
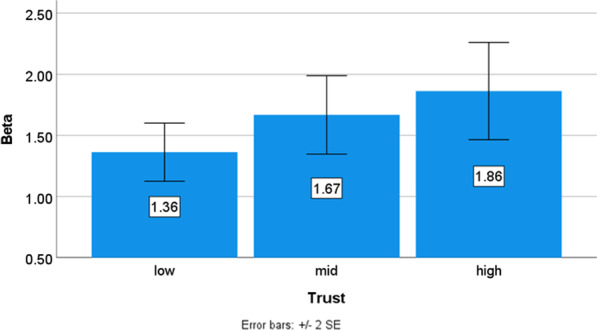
Average bias scores per level of trust in the authenticity of a banknote. When images were presented until response, a high trust has led to a more conservative bias, i.e. a lower tendency to declare a banknote a counterfeit. Error bars depict 95% confidence intervals

**Fig. 7:**
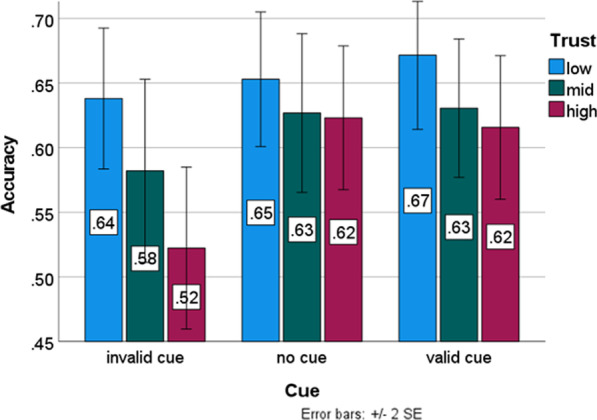
Average accuracy per level of trust (low, mid, high) and per cueing condition (invalid, no cue, valid). Presentation time is until response. Both a low trust in the authenticity (i.e. a high expectancy on the number of counterfeits) and valid cueing led to better performance. Error bars depict 95% confidence intervals

## Data Availability

The datasets used and/or analysed during the current study are available from the corresponding author on reasonable request.

## Appendix

### Replication experiment, presentation time increased

In line with our expectations, the 66 students, who were granted a longer presentation time, performed better than the panel. The average sensitivity for the no cue condition was 1.734, definitely higher than the sensitivity score for the CenTErdata panel of Dutch participants (0.386). This score is also considerably higher than a sensitivity score of 1.25, which is the norm that Raymond (2017) proposed for representing a reasonably good performance.

The influence of trust on the authenticity of banknotes was calculated with a GLM repeated measures. In this experiment higher trust influenced sensitivity scores negatively: *F*(2,66) = 4.103, *p* = 0.019 (Fig. 5). The bias of participants increased with a lower trust in the authenticity of banknotes: *F*(2,66) = 3.639, *p* = 0.029. This means that when the participants have low trust and expect a high ratio of counterfeits the criterion is also high. Such a bias is called conservative, i.e. not willing to make that much false alarms and taking the chance of lower hits. Conversely, a low expectancy on the number of counterfeits leads to a more liberal criterion, i.e. that participants made both more hits and false alarms. See Fig. 6. Just like the experiment with participants from the CenTErdata panel, we found main effects for accuracy per cueing validity (*F*(2,66) = 4.565, *p* = 0.012), η^2^*p* = 0.07 and trust (*F*(2,66) = 4.304, *p* = 0.015), *η*^2^*p* = 0.06 and no interaction between these factors: *F*(4,66) = 0.989, *p* = 0.414 (Fig. 7).
